# Announcement: 4th France Greece Italy Portugal Spain (FGIPS) Meeting in Inorganic Chemistry

**DOI:** 10.1155/MBD.1997.51

**Published:** 1997

**Authors:** Kevin A. Francesconi

**Affiliations:** Institute of Biology Odense University Odense M DK-5230 Denmark


					4TH FRANCE GREECE ITALY
PORTUGAL SPAIN (FGIPS) MEETING
IN INORGANIC CHEMISTRY
October 14 18, 1997
Corfu, Greece
HONORARY COMMITTEE
Marc JULIA, Socidtd Francaise de Chimie
Nikos KATSAROS, Association of Greek Chemists
George PNEUMATIKAKIS, Association of Greek Chemists
Ivano BERTINI, Italian Chemical Society
Romano CIPOLINI, National Research Council of Italy
Romao DIAS, A., National Research Council of Portugal
Formosinho SANCHES, A., Portuguese Chemical Society
Juan RODRIGUEZ-RENUNCIO, Spanish Chemical Society
Jose R. MASAGUER, Spanish Chemical Society
INTERNATIONAL SCIENTIFIC COMMITTEE
Gilbert G. A. BALAVOINE, France
Roger GUILARD, France
Nikos KATSAROS, Greece
Claudio BIANCHINI, Firenze, Italy
Antonio SGAMELLOTTI, Perugia, Italy
Jose MOURA, Portugal
Jose M. GONZALEZ-CALBET, Spain
Pablo ESPINET, Spain
Jacques LIVAGE, France
Nikos HADJILIADIS, Greece
Theofilos THEOPHANIDES, Greece
Giovani NATILE, Bari, Italy
Maria Jose CALHODRA, Portugal
Manuel ALMEIDA, Portugal
Virtudes MORENO, Spain
LOCAL ORGANIZING COMMITTEE
Nikos KATSAROS, NCSR "Demokritos" (Chairman)
George PNEUMATIKAKIS, Univ. of Athens Nick HADJILIADIS, Univ. of Ioannina
Nick KALOGERAKOS, Univ. of Athens Costas METHENITIS, Univ. of Athens
Costas MERTIS, Univ. of Athens Dimitris KESSISSOGLOU, Univ. of Salonika
Athanassios KOUTSOLELOS, Univ. of Crete Spyros PERLEPES, Univ. of Patras
CONFERENCE SECRETARIAT
Dr. Aglaia KOUTSODIMOU
NCSR "Demokritos", Inst. Physical Chemistry
GR-153 10 Ag. Paraskevi Attikis, GREECE
Tel: +30 1 6513111-9 ext 131, Fax: +30 1 6511766
e-mail' koutsod@cyclades.nrcps.ariadne-t.gr
SCIENTIFIC PROGRAM
The fourth French, Greek, Italian, Portuguese, Spanish meeting (4th FGIPS) will be held in Corfu,
Greece, from October 14 to October 18, 1997.
The symposium will resemble the previous GIPS meetings (Gandia 1990, Algarve 1992 and
Senigallia 1995) which were attended by two to four hundred scientists with expertise in:
Coordination and Bioinorganic Chemistry
Organometallic Chemistry and Catalysis
Solid State Chemistry and Materials
The program will emphasize cross disciplinary relations and promote interactions among participants
of the five different countries.
There will be plenary lectures, session lectures and minisymposia on the following topics:
1. Reaction Mechanisms
2. Metals in Medicine
3. Models for Metalloenzymes
4. Asymmetric Synthesis
5. Homogel)eous Catalysis
6. Stmctule and Reactivity of Organometallic Compounds
7. Magnetic, Electronic and Optical Properties of Materials
8. Surface Chemistry and Catalysis
9. Synthesis and Structural Characterization of Materials
10. Environnl ental Inorganic Chemistry
11 .Inorganic Chemistry and Nutrition
12. Theoretical Aspects in Inorganic Chemistry
Submission of Abstracts for contributed papers
Papers are welcome on research that is related to the cited topics.
Abstract forms and instructions will be given in the Second circular.
Registration fees will be 120USD for active participants and 80USD for accompanying persons. To
encourage attendance by young scientists, registration fees will be reduced to 80USD for graduate
students who present oral papers or posters.
Registration forms and instructions will also be given in the second circular.
Approximate fare for half board pension will be:
Hotel CHANDRIS
Half board, single room: 75USD
Half board, double room: 53USD per person
Conference will take place at the hotel CHANDRIS
Venue and Accommodation
The meeting will be held in Corfu (Greece) a beautiful island connected by sea and air to Athens. In
October the average temperature is about 20C. Accommodation will be available in convenient and
comfortable hotels located at the seashore and at walking distance from lecture halls.
Information on hotel accommodations, air and sea connections will be given in the second circular.
52

				

